# Resident interest and factors involved in entering a pediatric pulmonary fellowship

**DOI:** 10.1186/1472-6920-4-11

**Published:** 2004-07-26

**Authors:** William M Gershan

**Affiliations:** 1 Department of Pediatrics Section of Pulmonology Medical College of Wisconsin Milwaukee, Wisconsin 53226 USA

**Keywords:** Academic medicine, career paths, fellowship training, subspecialty practice.

## Abstract

**Background:**

Relatively little is known about interest in pediatric pulmonology among pediatric residents. The purpose of this study, therefore, was to determine at this institution: 1) the level of pediatric resident interest in pursuing a pulmonary fellowship, 2) potential factors involved in development of such interest, 3) whether the presence of a pulmonary fellowship program affects such interest.

**Methods:**

A questionnaire was distributed to all 52 pediatric residents at this institution in 1992 and to all 59 pediatric residents and 14 combined internal medicine/pediatrics residents in 2002, following development of a pulmonary fellowship program.

**Results:**

Response rates were 79% in 1992 and 86% in 2002. Eight of the 43 responders in 1992 (19%) had considered doing a pulmonary fellowship compared to 7 of 63 (11%) in 2002. The highest ranked factors given by the residents who had considered a fellowship included wanting to continue one's education after residency, enjoying caring for pulmonary patients, and liking pulmonary physiology and the pulmonary faculty. Major factors listed by residents who had not considered a pulmonary fellowship included not enjoying the tracheostomy/ventilator population and chronic pulmonary patients in general, and a desire to enter general pediatrics or another fellowship. Most residents during both survey periods believed that they would be in non-academic or academic general pediatrics in 5 years. Only 1 of the 106 responding residents (~1%) anticipated becoming a pediatric pulmonologist.

**Conclusions:**

Although many pediatric residents consider enrolling in a pulmonary fellowship (~10–20% here), few (~1% here) will actually pursue a career in pediatric pulmonology. The presence of a pulmonary fellowship program did not significantly alter resident interest, though other confounding factors may be involved.

## Background

The specialty of pediatric pulmonology is relatively new, having been recognized as a pediatric sub-specialty by the American Board of Medical Sub-specialties in 1984. In 1997, there were approximately 500 board certified pediatric pulmonologists in the United States and Canada [1]. This number has increased in recent years with 708 board certified pulmonologists being identified in 2003 [2]. It has been estimated that there is one pediatric pulmonologist for every 280,000 children in the United States [1]. There are over 50 pediatric pulmonary fellowship programs in North America with approximately 30–35 fellows graduating each year. The demand for pediatric pulmonologists has increased during the past decade, with many academic centers looking for two or more pulmonologists simultaneously [3]. A recent national survey of medical directors at children's hospitals across the country found that vacancy rates for faculty in pediatric pulmonology (25 of 136 positions vacant = 18.4%) was ranked second highest, behind only pediatric endocrinology, among over 40 pediatric subspecialties [3]. Despite this demand, little is known about overall interest in this field among pediatric residents. For this reason, this study was completed to examine interest among pediatric residents in entering a pulmonary fellowship. The specific aim of this study, therefore, was to determine at our institution: 1) the level of pediatric resident interest in pursuing a pulmonary fellowship; 2) potential factors involved in development of such interest; 3) whether the presence of an active fellowship program affected resident interest in such a program.

## Methods

This study involved the distribution of a questionnaire to all pediatric residents. The questionnaire was initially distributed in 1992 prior to institution of a pulmonary fellowship. The questionnaire was placed in the hospital mailbox of each resident. The study was repeated (and the questionnaire redistributed) in 2002 after the fellowship, which began in 1994, had been functioning for several years. To improve the response rate, the questionnaire was distributed twice, one month apart, during each time period. The questionnaire was a three-page, 18 question form that took approximately 15 minutes to complete (copy enclosed in Appendix [see Additional file 1]). The questionnaire asked several epidemiological questions (e.g. year of residency, whether medical school was attended at this institution), several questions that pertained to an individual resident's interest in entering any specialty fellowship, and approximately 12 questions specifically dealing with interest in doing a pulmonary fellowship and factors that may be either positively or negatively related to such interest. The survey utilized a flow diagram, and consequently, slightly different questions were asked depending on a resident's interest or lack of interest in a pulmonary fellowship. The study was analyzed after dividing the respondents into 2 groups: those that had considered a pulmonary fellowship during their residency ("+PF") and those that had not considered a pulmonary fellowship during their residency ("-PF"). Neither Human Subjects approval nor specific resident consent was obtained. However, all questionnaires were answered anonymously and residents were in no way coerced or forced to complete the survey. Residents who completed the form were given a certificate for a free meal at Children's Hospital of Wisconsin. Several questions were based on a 4-point Likert scale and ranked from 0 ("not at all important") to 3 ("very important") in a resident's decision to consider or not consider doing a pulmonary fellowship. Numerical responses for each survey period were averaged (mean ± SD) and ranked. Fisher's exact test was used to compare categorical variables and the Student's t-test was used to compare the means of measured variables in 2 independent samples. A p value of ≤ 0.05 was considered significant. Medical Education records were also reviewed anonymously (data collated by year of residency) in 2003 to determine residents' actual career decisions.

## Results

The questionnaire was distributed to 52 pediatric residents (including 2 chief residents) in 1992 and to 59 pediatric residents (including 3 chief residents) and 14 medicine/pediatric residents (including 1 chief resident) in 2002. A medicine-pediatric residency program did not exist during the initial distribution period. To avoid compromising confidentiality in the relatively small medicine/pediatric group, residents were not asked to list their residency program in 2002 and consequently the 2 resident groups during that period were combined. Forty-three of the 52 residents completed the survey in 1992 (79%) compared to 63 of 73 (86%) during 2002 (p = NS).

Of the 43 respondents in 1992, 30 (70%) had considered doing a fellowship in any pediatric subspecialty and of those, 15 (35% of all respondents) believed they were "very likely" to do a fellowship. Of the 63 respondents in 2002, 40 (63%) had considered doing a pediatric fellowship and 16 of those (25% of all respondents) were "very likely" to continue with fellowship training. These numbers compare with 9 of the 52 residents in 1992 (17%) who actually completed fellowship training compared to 16 of the 47 graduating residents from the 2002 survey (34%) who began fellowship training in 2003 or 2004.

Eight residents (19%) in 1992 had considered a pulmonary fellowship compared to 7 residents (11%) in 2002 (p = NS). Table 1 lists these responses based on survey period and year of residency (internal medicine-pediatric residency is 4 years long). Although all residents have significant exposure to pulmonary patients during their residency, no correlation existed between interest in a pulmonary fellowship and having previously taken a one-month elective in pediatric pulmonology during residency. Three of the 15 residents who had considered a pulmonary fellowship ("+PF") had taken a pulmonary elective compared to 7 of the 91 residents who had not considered such a fellowship ("-PF"; p = 0.15). Of the 10 residents who had taken a pulmonary elective, only 2 thought that the elective experience affected their decision in considering a pulmonary fellowship (one from the +PF and one from the -PF group).

**Table 1:** Resident response based on year of survey (1992 or 2002) and year of residency (1–5)

1992: Residency Year	Resident Number	Number Responded	% Response	+PF*	-PF**
1	21	20	95%	5	15
2	19	12	63%	2	10
3	10	9	90%	0	9
4	2	2	100%	1	1
1992 totals	52	43	79%	8	35
2002: Residency Year	Resident Number	Number Responded	% Response	+PF*	-PF**

1	24	22	92%	3	19
2	21	20	95%	3	17
3	22	17	77%	1	16
4	5	3	60%	0	3
5	1	1	100%	0	1
2002 totals	73	63	86%	7	56

* +PF refers to those residents who had considered doing a pulmonary fellowship during their residency. ** -PF refers to those residents who had not considered doing a pulmonary fellowship during their residency.

Table 2 lists and ranks the reasons given by residents who had considered doing a pulmonary fellowship. The highest ranked factors given by +PF residents included wanting to continue one's education after residency, enjoying caring for pulmonary patients in general, and enjoying both pulmonary physiology and the pulmonary faculty. The only factor that approached a statistically significant difference for +PF between the 2 survey periods was the statement "I enjoy the tracheostomy-ventilator population," with a score of 0.6 ± 0.5 in 1992 vs. 1.6 ± 1.1 in 2002 (p = 0.057), suggesting that this factor became somewhat more important to the 2002 +PF residents in their consideration of a pulmonary fellowship. Table 3 lists and ranks reasons given by residents who had not considered doing a pulmonary fellowship. The scores given by -PF residents were generally not as high as those given by +PF residents, i.e. the factors listed were not as important to the -PF vs. the +PF residents. Major factors listed by -PF residents included not enjoying the tracheostomy/ventilator population and certain pulmonary patients in general, including chronic patients, as well as a desire to enter general pediatrics or another fellowship. The significant differences for -PF between the 2 survey periods are listed in Table 3.

**Table 2:** Mean (± SD) scores for factors given by +PF residents (those who considered a pulmonary fellowship)

Factor*	1992 Score	1992 Rank	2002 Score	2002 Rank
I want to continue my education after residency	2.8 ± 0.4	1	2.4 ± 0.8	1
I enjoy pulmonary-related procedures	2.5 ± 0.8	2	2.0 ± 1.0	10
I enjoy caring for pulmonary patients	2.4 ± 0.5	3	2.4 ± 0.5	1
I like pulmonary physiology	2.4 ± 0.8	3	2.3 ± 0.8	4
I like the pulmonary faculty	2.4 ± 0.5	3	2.4 ± 0.8	1
I enjoy pulmonary inpatient coverage	2.3 ± 0.5	6	2.3 ± 0.5	4
I enjoyed working with a particular faculty member	2.1 ± 0.9	7	2.1 ± 0.7	7
I might enjoy pulmonary-related research	2.0 ± 1.2	8	1.4 ± 0.8	12
I enjoy pulmonary clinics	2.0 ± 1.0	8	2.2 ± 0.8	6
I enjoy cystic fibrosis patients	1.9 ± 1.1	10	2.1 ± 1.1	7
I enjoyed caring for a particular pulmonary patient	1.7 ± 1.3	11	2.1 ± 0.7	7
The salary would be attractive	1.1 ± 1.2	12	1.0 ± 0.8	13
I enjoy the tracheostomy/ventilator population	0.6 ± 0.5	13	1.6 ± 1.1#	11

*Each factor was scored from 0 ("not at all important") to 3 ("very important") relating to a resident's decision to consider doing a pulmonary fellowship. #2002 score approached statistically significant difference vs. 1992 score (p = 0.057).

**Table 3:** Mean (± SD) scores for factors given by -PF residents (those who did not consider a pulmonary fellowship)

Factor*	1992 Score	1992 Rank	2002 Score	2002 Rank
I don't enjoy the trach/vent population	2.0 ± 1.1	1	1.9 ± 1.3	1
I want to enter general pediatrics	1.9 ± 1.2	2	1.8 ± 1.3	2
I don't enjoy certain pulmonary patients	1.6 ± 1.1	3	1.2 ± 1.1	4
There are too many chronic patients	1.5 ± 1.1	4	1.5 ± 1.1	3
I want to enter another fellowship	1.5 ± 1.2	4	1.2 ± 1.3	4
I don't know enough about it to decide	1.5 ± 1.1	4	1.0 ± 1.1	7
Not enough pulmonary patient experience	1.4 ± 1.1	7	0.6 ± 0.9#	9
I don't enjoy the BPD^+ ^population	1.2 ± 1.0	8	1.2 ± 1.1	4
I don't think pulmonary is very interesting	0.9 ± 0.9	9	0.6 ± 0.8	9
Some of the pulmonary patients scare me	0.7 ± 0.8	10	0.8 ± 1.0	8
I can't afford being a fellow	0.7 ± 1.1	10	0.4 ± 0.8	12
The pulmonologists work too hard	0.4 ± 0.6	12	0.4 ± 0.7	12
I don't enjoy the cystic fibrosis population	0.3 ± 0.6	13	0.6 ± 0.9	9
Too few pulmonary job openings	0.3 ± 0.5	13	0.0 ± 0.2#	17
I don't enjoy the asthma population	0.2 ± 0.5	15	0.4 ± 0.8	12
Pulmonologists don't earn enough money	0.2 ± 0.5	15	0.1 ± 0.4	16
Poor experiences with pulmonary faculty	0.2 ± 0.4	15	0.3 ± 0.8	15

*Each factor was scored from 0 ("not at all important") to 3 ("very important") relating to a resident's decision to not consider doing a pulmonary fellowship. #Indicates 2002 score significantly different than 1992 score, p < 0.001. ^+ ^bronchopulmonary dysplasia

The last question in the survey asked residents what they thought they would be doing 5 years in the future. These responses are shown in Table 4. The majority of residents during both survey periods believed that they would be in either non-academic or academic general pediatrics in 5 years. However, when grouped together during the 2 periods, the +PF residents were less likely to see themselves in the future as general pediatricians compared to the -PF residents (p < 0.05). Interestingly, when the actual career decisions of the 1992 residents were reviewed, 35 of the 52 residents (67%) went into non-academic general pediatrics, 5 (10%) entered academic general pediatrics, 9 (17%) completed pediatric subspecialty training, 2 (4%) began a non-pediatric medical specialty, and in 1 case (2%) the eventual career decision could not be determined. Only 1 resident (from 1992 survey) in the entire group of 106 survey responders (~1%) believed they would be in the field of pediatric pulmonology in the future. This resident did complete a pulmonary fellowship at another institution and is currently in an academic pulmonary practice. Additionally, a second resident (from 1992 survey) is currently an academic pediatric pulmonologist after having initially completed one year of a different subspecialty fellowship following residency. That resident then completed a pulmonary fellowship at this institution. Lastly, there was no significant difference in the overall level of interest in a pulmonary fellowship from 1992 compared to 2002.

**Table 4:** Anticipated professional plans of resident 5 years following survey completion

Category	1992		2002	
	+PF	-PF	+PF	-PF

General pediatrics, non-academic	1	14.5*	2	29
Academic general pediatrics	1	5	1	8.5
Academic non-pulmonary pediatric specialty	3	12	2	16
Academic pediatric pulmonology	1	0	0	0
Non-academic non-pulmonary pediatric specialty	1	1.5	1	1.5
Non-academic pediatric pulmonology	0	0	0	0
Non-pediatric medical specialty	0	0	0	1
Non-medical vocation	0	0	0	0
Unknown	1	2	1	0

See text and footnote to Table 1 for explanation of +PF and -PF. The numbers represent actual number of residents responding. *Some residents listed 2 categories, hence their score was divided between them.

## Discussion

This study found that a significant percentage of pediatric residents considered doing a pulmonary fellowship after their residency training, ranging from 11% in the 2002 group to 19% in the 1992 group. However, these residents also viewed themselves as less likely to enter general pediatrics perhaps suggesting that they were simply considering several pediatric subspecialties at some time during their residency training. This seems likely, as the highest scored factor by the +PF residents was the desire to continue their education after residency. Despite fairly high percentages of residents considering a pulmonary fellowship, only 1 resident in the entire group (~1%) actually believed that they would be a pediatric pulmonologist 5 years after the survey was completed. This percentage is very similar to that of first-time takers of the 1995 General Pediatrics Certifying Examination who believed they would be a pediatric pulmonologist in the future (1.1%) [4]. If one were to extrapolate this number to the entire class of graduating pediatric residents per year, about 2600 residents, approximately 30 residents would be entering the field of pediatric pulmonology each year [5]. This number is very similar to the actual number of graduating residents who enter a pediatric pulmonary fellowship each year [2].

The majority of residents who considered doing a pulmonary fellowship (8 of 15) were in their first year of residency. From personal experience, residents often consider various practice options early on in their training and frequently do not tend to narrow their choices until their second or third year of residency. This observation should be kept in mind when trying to recruit residents for pulmonary fellowship positions by seeking out those residents potentially interested in Pulmonology early on in residency rather than later.

The ranking of factors that may contribute to an interest in a pulmonary fellowship were remarkably similar during the 2 time periods. The only score that approached a statistically significant difference was the statement "I enjoy the tracheostomy/ventilator population" and this score tended to increase in 2002. However, a dislike for the tracheostomy/ventilator population also received the highest score among those residents not interested in a pulmonary fellowship and was even higher than both the desire to enter general pediatrics and another fellowship program. These data may simply be a center phenomenon but might suggest a more global "disinterest" in this patient population that may need to be further studied. Two scores in the -PF resident group decreased from 1992 to 2002: not enough pulmonary patient experience to decide on a pulmonary fellowship and too few perceived pulmonary job openings. The first may be a positive reflection on the local resident experience with pulmonary patients in recent years or on the fellowship program itself though this is only speculative. The second received very low scores during both periods and may not be very relevant. However, there has been some evidence of a reversal in the prior trend of residents entering general pediatrics in recent years, suggesting greater interest among residents in fellowships [6]. On the other hand, a recent survey of practicing pediatric pulmonologists noted that 69% of respondents did not believe that there was need for additional pulmonologists in their locale [7]. Interestingly, despite recent articles relating high post-residency debt with a disinterest in entering a fellowship, this was not noted in this study with mean scores during both survey periods of less than 1.0 for the statement "I can't afford being a fellow." [8,9]. This study did not ask residents to state their current level of indebtedness. Other "job concern" factors including job availability and the workload of pulmonologists did not appear to be significant negative factors for the -PF residents.

This study has certain shortcomings. This study asked residents to score specific factors that may or may not have been relevant to an individual resident. Although residents were given the opportunity to add personal comments, few did. In addition, certain patient populations, e.g those with respiratory infections, were not included as options in the survey and these omissions could cause study bias. Other factors that may contribute to residents' decisions regarding fellowship training were not addressed in this study. These factors include resident teaching by the faculty, resident gender, spouse occupation, mentor encouragement or other personal reasons [10-12]. In addition, this study involved residents in only one program and cannot be generalized to all residency programs. Although this study did not find that the presence of a pulmonary fellowship significantly affected resident interest in such a fellowship, a significant difference may have been found with a larger sample size. In fact, the study appears to be underpowered despite the high response rate. More than 250 individuals would need to have been included in the study to detect a significant difference in residents' initial interest in a pulmonary fellowship (assuming a power of 80%). In addition, other changes may have occurred within the residency program including higher loan repayments, changes in department philosophy (e.g. new department chairman), and changes in local pediatric job opportunities, which may have affected the results. Despite these limitations, this study may prove useful to those who are recruiting pediatric residents as potential pulmonary fellows. Further larger studies looking at multiple residency programs may provide more insight in the future. Lastly, studies like these help to reiterate the need for the specialty of pediatric pulmonology to "prospectively and objectively determine realistic future training needs" [13].

## Conclusions

Although many pediatric residents consider enrolling in a PF (~10–20% here), few (~1% here) will actually pursue a career in pediatric pulmonology. The presence of a PF program did not significantly alter resident interest, though other confounding factors may be involved.

## Abbreviations

BPD: bronchopulmonary dysplasia

PF: pulmonary fellowship

+PF: those residents who considered taking a pulmonary fellowship

-PF: those residents who had not considered taking a pulmonary fellowship

## Competing interests

None declared.

## Pre-publication history

The pre-publication history for this paper can be accessed here:



## Supplementary Material

Additional File 1 Appendix. Resident questionnaire Click here for file
