# Supplementary material for: Resident interest and factors involved in entering a pediatric pulmonary fellowship
Source: BMC Med Educ. 2004 Jul 26;4:11. doi: 10.1186/1472-6920-4-11 (PMC503396; doi:10.1186/1472-6920-4-11)
Supplement: Additional File 1 — Appendix. Resident questionnaire [file 1472-6920-4-11-S1.doc]

**Appendix**

MEMO: REGARDING RESIDENT INTEREST IN A PULMONARY FELLOWSHIP

DATE: NOVEMBER 18, 2002

TO: ALL PEDIATRIC AND MED/PEDS RESIDENTS

FROM: WILLIAM GERSHAN, M.D.

FELLOWSHIP DIRECTOR, PEDIATRIC PULMONOLOGY

This is a brief questionnaire regarding pediatric residents' interests in a pulmonary fellowship. Its purpose is twofold - first, to help identify residents who may be interested in a pulmonary fellowship in the future, and second, to identify factors that may influence your decision in becoming a fellow. I greatly appreciate your time in filling out this questionnaire and would be happy to talk with you if you have any further questions. This survey is strictly anonymous and your comments are welcome. I have enclosed a meal ticket as a token of my appreciation for your time and effort.

1) In which year of residency are you? Please check one response.

first second third fourth___ fifth___

2) Did you attend medical school here? yes no ­­___

3) Have you considered doing a fellowship in a pediatric subspecialty? yes no___ which one? ______________________

4) At this time, how likely are you to do a fellowship?

very likely somewhat likely not likely ___

5) Have you considered doing a pediatric pulmonary fellowship?

yes no If no, skip to #7.

6) Please rank the following statements as to their importance in your consideration of doing a pulmonary fellowship. Please rank each statement using the following scale:

0 = not at all important

1 = mildly important

2 = moderately important

3 = very important

___ I enjoy caring for pulmonary patients, in general

___ I enjoy cystic fibrosis patients

___ I enjoy the tracheostomy/ventilator population

___ I enjoy pulmonary clinics

___ I enjoy pulmonary inpatient coverage

___ I enjoy pulmonary-related procedures

___ I think I might enjoy pulmonary-related research

___ I like the pediatric pulmonary faculty

___ I enjoyed working with a particular faculty member

___ I like pulmonary physiology

___ I enjoyed caring for a particular pulmonary patient

___ I want to continue my education

___ The salary would be attractive

___ Other(s) - please write in ____________________________________

____________________________________

If you answered #6, skip to #8.

7) Please rank these reasons 0-3 (0 = not important to 3 = very important) as to why you answered no to #5:

___ I want to go into general pediatrics

___ I want to enter a different fellowship program

___ I can't afford being a fellow

___ I don't think pulmonary is very interesting

___ I haven't had enough pulmonary patient experience

___ The pulmonologists work too hard

___ I don't enjoy certain pulmonary patients

___ e.g. cystic fibrosis

___ e.g. tracheostomy/ventilator

___ e.g. asthma

___ e.g. bronchopulmonary dysplasia

___ Some of the pulmonary patients scare me

___ Too many chronic patients

___ I don't know enough about pulmonary now to decide

___ I have not had good experiences with the pulmonologists

___ There are too few job openings in pediatric pulmonology

___ Pulmonologists do not earn enough money

___ Other(s):

8) Have you taken a pulmonary elective here?

yes no If no, skip to #15.

9) Was the elective a good experience?

yes no If yes, skip to #11.

10) Why wasn't the elective a good experience? Mark all that apply.

___ Not enough teaching

___ Too much work

___ Too little work

___ Not enough outpatient experience

___ Too much responsibility

___ Not enough responsibility

___ Not enough help from pulmonary faculty

___ Patients were not interesting

___ Patient population not diverse enough

___ The pulmonary faculty did not treat me well

___ Other(s):_________________________________________________

If you answered #10, skip to #12.

11) Why was the elective a good experience? Mark all that apply.

___ Good teaching

___ Interesting patients

___ I was able to perform procedures

___ Good combination of various responsibilities

___ I was treated well by pulmonary faculty

___ I had time to learn

___ Other(s):_________________________________________________

12) Would you recommend the pulmonary elective to other residents?

yes no ___

13) Did the pulmonary elective affect your decision to consider

doing a pulmonary fellowship?

yes no If no, skip to #17

14) Please rate the following factors 0-3 as to how much they

affected your decision to do a pulmonary fellowship (0 = not

important, 3 = very important).

___ Teaching during elective

___ Patient population

___ Contact with pulmonologist(s)

___ Contact with patient families

___ Amount of work

___ Variety of work

___ Interest shown you by others Please skip to #17.

15) Do you think that you will take a pulmonary elective here?

yes no If yes, skip to #17.

16) If the answer to #15 is no, please mark the reason(s) why:

___ I don't have enough elective time

___ Pulmonary residents work too hard

___ I don't like pulmonary

___ I have not heard good things about the elective

___ There are other electives that I would prefer to take

___ I already see enough pulmonary patients

___ I don't like one or more of the pulmonary attendings

17) ”If I did eventually decide to do a pulmonary fellowship, I would do it somewhere else.” If this statement is true, please mark the reason(s) why you feel this way:

___ It's important to go somewhere else for more training

___ I would go somewhere else for a different perspective

___ I don't think that I would receive good training here

___ I'm tired of this city and/or its weather

___ I'm tired of this medical school/hospital/institution

___ I don't like the pulmonary faculty

___ I need to go back home for one or more reasons

___ Fellowship here is too new

___ Other(s): _______________________________________________

18) What do you think you will be doing 5 years from now? Please list 1 response only.

___ General pediatrics, non-academic

___ Academic general pediatrics

___ Academic non-pulmonary pediatric subspecialty (list, if known ______________________)

___ Academic pediatric pulmonology

___ Non-academic, non-pulmonary pediatric subspecialty (list, if known ______________________)

___ Non-academic pediatric pulmonology

___ Non-pediatric medical specialty

___ Non-medical vocation

Thank you again for taking time to fill out this form. It will certainly help us plan for the future and will hopefully improve the pulmonary elective. Please return the form in the enclosed envelope and contact me if you have any questions.
